# Effects of intrinsic tannins on proteolysis dynamics, protease activity, and metabolome during sainfoin ensiling

**DOI:** 10.3389/fmicb.2022.976118

**Published:** 2022-08-18

**Authors:** Rong Zheng Huang, Xuzhe Wang, Chunhui Ma, Fanfan Zhang

**Affiliations:** Grassland Science, School of Animal Technology, Shihezi University, Shihezi, China

**Keywords:** sainfoin, silage, metabolites, proteolysis, protease

## Abstract

Condensed tannins (CT) from sainfoin have a high capacity to inhibit proteolysis. A previous study reported that CT from sainfoin can inhibit lactic acid bacteria activity and decrease ammonium-nitrogen (N) content during sainfoin ensiling; however, no study has focused on the metabolome of ensiled sainfoin. The objective of the present study was to investigate the effects of CT [following supplementation of deactivated CT with polyethylene glycol (PEG)] on protease activity, keystone bacteria, and metabolome during sainfoin ensiling. According to the results, PEG amendment increased non-protein N, amino acid, and soluble protein contents significantly (in the 49.08–59.41, 116.01–64.22, and 23.5–41.94% ranges, respectively, *p* < 0.05) during ensiling, whereas neutral detergent-insoluble protein and acid detergent-insoluble protein were decreased significantly (in the 55.98–64.71 and 36.58–57.55% ranges, respectively, *p* < 0.05). PEG supplementation increased aminopeptidase and acid protease activity after 3 days of ensiling (*p* < 0.05) and increased carboxypeptidase activity during the entire ensiling process (*p* < 0.05). The keystone bacteria changed following PEG addition (*Stenotrophomona*s, *Pantoea*, and *Cellulosimicrobium* in the control vs. *Microbacterium, Enterococcus*, and *Brevundimonas* in the PEG-treated group). In total, 510 metabolites were identified after 60 days of sainfoin ensiling, with 33 metabolites annotated in the Kyoto Encyclopedia of Genes and Genomes database. Among the metabolites, phospholipids were the most abundant (72.7% of 33 metabolites). In addition, 10 upregulated and 23 downregulated metabolites were identified in the PEG-treated group when compared with the control group, after 60 days of ensiling (*p* < 0.05). *Pediococcus* (correlated with 20 metabolites, *R*^2^ > 0.88, *p* < 0.05) and *Lactobacillus* (correlated with 16 metabolites, *R*^2^ > 0.88, *p* < 0.05) were the bacteria most correlated with metabolites. The results suggested antagonistic effects between *Lactobacillus* and *Pediococcus* during ensiling. The decreased proteolysis during sainfoin ensiling was mainly attributed to the inhibition of protease activity by CT, particularly carboxypeptidase activity. In addition, proteolysis decreased partly due to CT inhibiting *Pediococcus* activity during ensiling, with *Pediococcus* being significantly and positively correlated with dopamine after 60 days of ensiling (*R*^2^ = 0.8857, *p* < 0.05).

## Introduction

During ensiling, especially in the case of legumes with high protein contents, proteolysis occurs driven by plant enzymes and microbial activity, which leads to the production of high non-protein nitrogen (NPN) contents (McDonald et al., 1991). During rumen microbial protein synthesis, less NPN from silage is utilized than NPN from fresh forages (Wetherall et al., 1995). Several studies have observed that condensed tannins (CT) reduce protein degradation during ensiling, due to the formation of stable compounds following the binding of CT to protein and inhibition of microbial activity (Peng et al., 2018; He et al., 2020). CT are plant secondary metabolites that are widely distributed in numerous plants, such as sericea lespedeza (*Lespedeza cuneata* Dum.-Cours.), crown vetch (*Coronilla varia* L.), hedysarum (*Hedysarum alpinum* L.), birdsfoot trefoil (*Lotus corniculatus* L.), and sainfoin (*Onobrychis viciifolia*). Among the plants above, CT from sainfoin is favored considering it exhibits the highest capacity to bind protein, and inhibits cellulose digestion by rumen bacteria the least so that it delivers a greater amount of protein to the abomasum without adverse effects on rumen microbial activity (McAllister et al., 2005).

Sainfoin is widespread in North America, Europe, and the Middle East, as a perennial forage and fodder legume (Carbonero et al., 2011). CT from sainfoin reportedly reduces protein degradation (Aufrere et al., 2014), reduces methane production (Wang et al., 2021), and has antihelmintic potential against gastrointestinal nematodes (Novobilsky et al., 2013) in the rumen, which improves animal performance.

During alfalfa ensiling, NPN contents decreased with an increase in sainfoin application rate, which was attributed to the CT in sainfoin inhibiting protein degradation (Wang et al., 2021). Similar results have been observed by other researchers when polyethylene glycol (PEG) is used to minimize the effect of CT from sainfoin (Peng et al., 2018; He et al., 2020). However, to the best of our knowledge, few researchers have explored the effects of CT from sainfoin on protease activity and the metabolome during sainfoin ensiling (Theodoridou et al., 2012). A previous study found that CT inhibits *Pediococcus* activity without affecting *Enterobacter* activity. *Enterobacter* can produce amino acid nitrogen during ensiling; however, CT decreases amino nitrogen (AN) contents during the entire ensiling process (Huang et al., 2022). Considering the protein degradation rate is correlated with plant enzyme and microbial activity, CT of sainfoin could have strong effects on protease activity. However, considering the relative abundance of *Enterobacter* has been reported to be 1.04–17.96% during sainfoin ensiling (Huang et al., 2022), the relationship between microbial activity and protein degradation remains unclear. Consequently, profiling the silage metabolome could enhance our understanding of the biological processes underlying silage fermentation (Guan et al., 2020). Therefore, the present study aimed to investigate the effects of CT on protease activity and the sainfoin metabolome during sainfoin ensiling.

## Materials and methods

### Forage and silage preparation

Sainfoin (*O. viciifolia*) was planted in Shihezi City, Xinjiang Province, China. Whole-plant sainfoin was harvested in July 2021 at the early flower stage. After wilting for 12 h to a dry matter (DM) content of approximately 240 g/kg fresh weight, the samples were chopped into 1— to 2-cm pieces. The samples were then sprayed with a solution of 640 g/l PEG (Sigma, molecular weight, 6,000) at a rate of 217 ml/kg DM to achieve a CT:PEG ratio of 1:2 in the samples (Peng et al., 2018). According to Jones and Mangan (1977), PEG inactivates condensed tannins’ biological activity due to its specific form of stable PEG-CT complexes attributed to binds with CT by H-bonds. Three separate piles were prepared and treated separately and bagged randomly for the control and PEG-treated groups. The control samples were sprayed with an equivalent amount of distilled water. After spraying, 1,000 g samples of both the chopped treated and control sainfoin were packed into polyethylene plastic bags (30 × 50 cm), then compacted and sealed using a vacuum sealer. Three replicates were prepared for the control and treatment samples. The bags were stored indoors at 23°C. After ensiling, samples were obtained after different ensilage days (3, 7, 14, 30, and 60) and stored at −20°C for later analyses.

### Characteristics of ensiled sainfoin

Silage samples from separate bags for 3 (five fermentation days) × 2 (two treatments) × 3 (replicates) of each silage treatment were collected. Silage samples were dried at 65°C for 48 h and ground using a 1.0-mm in preparation for DM content determination. The NPN, neutral detergent-insoluble protein (NDIP), and acid detergent-insoluble protein (ADIP) concentrations were analyzed according to Licitra et al. (1996). Soluble protein (SOLP) concentrations were analyzed according to Bradford and Marion (1976). The protein fraction dynamics during sainfoin ensiling were analyzed according to the Cornell Net Carbohydrate and Protein System (CN) by Sniffen et al. (1992).

### Proteolytic enzyme activity analysis

Samples (10 g) were obtained from each silage treatment, blended in a homogenized (L-1BA, Kuansons Biotechnology Co. Ltd., Shanghai, China) 50 ml of 0.1 M sodium phosphate buffer (pH 6.5), and then centrifuged at 10,000 × *g* for 10 min at 4°C. The supernatant was stored at −80°C until proteolytic enzyme activity analysis. L-leucine-p-nitroanilide was used as the substrate for aminopeptidases (APs), N-carbobenzoxy-L-phenylalanine-L-alanine was used as the substrate for carboxypeptidase (CP), and azocasein was used as the substrate for acid protease (ACP). Proteolytic enzyme activity analysis was carried out according to Guo et al. (2007).

### Metabolite analysis

#### Sample preparation and metabolite extraction

After 60 days of ensiling, 50-g samples of the ensiled sainfoin treatments were obtained and metabolites extracted using 400-μl methanol:water (4:1, v/v) solution. The mixture was allowed to settle at −20°C and treated using a high throughput tissue crusher (Wonbio-96c Shanghai Wanbo Biotechnology Co., Ltd., Shanghai, China) at 50 Hz for 6 min, followed by vortexing for 30 s and ultrasonication at 40 kHz for 30 min at 5°C. Afterward, the samples were placed at −20°C for 30 min to precipitate proteins. After centrifugation at 13,000 × *g* at 4°C for 15 min, the supernatant was collected for ultra-high performance liquid chromatography-tandem mass spectrometry (UHPLC-MS/MS) analysis.

#### Ultra-high performance liquid chromatography-tandem mass spectrometry analysis

Chromatographic separation of the metabolites was performed on a Thermo UHPLC system equipped with an ACQUITY BEH C18 column (100 mm × 2.1 mm i.d., 1.7 μm; Waters, Milford, MA, United States). The mobile phases consisted of 0.1% formic acid in water (solvent A) and 0.1% formic acid in acetonitrile:isopropanol (1:1, v/v) solution (solvent B). The solvent gradient was as follows: from 0 to 3 min, 95% (A):5% (B) to 80% (A):20% (B); from 3 to 9 min, 80% (A):20% (B) to 5% (A):95% (B); from 9 to 13 min, 5% (A):95% (B) to 5% (A):95% (B); from 13 to 13.1 min, 5% (A):95% (B) to 95% (A):5% (B), from 13.1 to 16 min, 95% (A):5% (B) to 95% (A):5% (B) for equilibrating the systems. The sample injection volume was 2 μl and the flow rate was 0.4 ml/min. The column temperature was maintained at 40°C. The mass spectrometric data were collected using a Thermo UHPLC-Q Exactive Mass Spectrometer equipped with an electrospray ionization (ESI) source operating in either positive or negative ion mode. The optimal conditions were set as follows: Aus gas heater temperature, 400°C; Sheath gas flow rate 40 psi; Aus gas flow rate 30 psi; ion-spray voltage floating (ISVF), −2,800 V in negative mode and 3,500 V in positive mode, respectively; normalized collision energy, 20–40–60 V rolling for MS/MS. Data acquisition was performed in the Data Dependent Acquisition (DDA) mode. The detection was carried out over a mass range of 70–1,050 m/z.

#### Metabolite analysis

Multivariate statistical analysis was performed using the ropls package (v1.6.2^1^) in R on the Majorbio Cloud Platform.^2^ Principal component analysis (PCA) based on an unsupervised method was applied to obtain an overview of the metabolic data. General clustering, trends, or outliers were visualized. All the metabolite variables were scaled to unit-variances before conducting the PCA. Orthogonal partial least squares discriminate analysis (OPLS-DA) was used for statistical analysis to determine global metabolic changes between comparable groups. All the metabolite variables were subjected to Pareto Scaling before the OPLS-DA. The model validity was evaluated based on model parameters *R*^2^ and *Q*^2^, which provide information on interpretability and predictability, respectively, of the model, and minimize over-fitting risk. Variable importance in projection (VIP) was calculated using the OPLS-DA model. The *p*-values were estimated using paired Student’s *t*-test on single-dimensional statistical analysis.

Statistical significance among groups was identified with VIP values >1 and *p* < 0.05. The Majorbio Cloud Platform (Majorbio Bio-pharm Technology Corporation, Shanghai, China^3^) was used for further analyses.

### Statistical analysis

The sainfoin silage data were subjected to a two-way analysis of variance, 2 × 5 factorial Complete Randomized Design (PEG and control treatments × five ensiling times days). Data were analyzed using IBM SPSS 22 Statistics (IBM Corp., Armonk, NY, United States). Significant differences between treatments were determined using Tukey’s test at *p* < 0.05. The ammonium nitrogen (NH_4_^+^-N), cured protein (CP), and bacteria sequence (SRA number for bacteria: PRJNA796365) data used were obtained from our former study (Huang et al., 2022).

## Results

### Nitrogen distribution in sainfoin silage

Nitrogen distribution during sainfoin ensiling is listed in Table 1. SOLP content decreased in both the control and treated groups with the prolongation of ensiling; SOLP content in the control decreased significantly after 7 days of ensiling (8.96 g/kg DM vs. 5.71 g/kg DM, *p* < 0.05), then stabilized with the prolongation of ensiling; SOLP content was the lowest in the PEG-treated group after 14 days of ensiling, which was 7.63 g/kg DM. However, the control had the lowest SOLP content during the entire ensiling process (*p* < 0.05). Similar results were observed for the effect of ensiling time on NDIP and ADIP contents. PEG addition decreased NDIP and ADIP contents markedly during ensiling by up to 100%, when compared with the contents in the control (*p* < 0.05). NPN and SOLP content trends in the control were similar. In addition, PEG supplementation increased NPN content significantly during the entire ensiling process (*p* < 0.05), by up to 59.41%, when compared with the contents in the control after 60 days of ensiling. Ensiling time increased amino acid (AA) contents in both the control and PEG-treated groups (*p* < 0.05), by 16.43 and 35.50%, respectively, after 60 days of ensiling. Furthermore, the PEG-treated group had the highest AA contents during the entire ensiling process (*p* < 0.05), which were up to twofold the contents in the control group.

**TABLE 1:** Effects of intrinsic tannins on nitrogen distribution in sainfoin silage^1^.

Item	Treatment	Days of ensiling					SEM	*P*-value		
		3	7	14	30	60		Day	PEG	D × P
CP (g/kg DM)	CK	240.13Aa	239.64Aa	240.85Aa	244.70Aa	246.79Aa	2.72	<0.01	<0.01	<0.01
	PEG	219.60Ba	202.90Bb	204.52Bb	216.02Bac	208.02Bbc				
SOLP (g/kg DM)	CK	8.96Ba	5.71Bb	5.82Bb	6.70Bb	6.08Bb	0.276	<0.01	<0.01	0.01
	PEG	11.52Aa	9.37Ab	7.63Ac	9.00Abc	8.63Abc				
NDIP (g/kg DM)	CK	110.44Aa	88.06Ab	85.33Ab	87.28Ab	78.02Ac	4.05	<0.01	<0.01	<0.01
	PEG	55.68Ba	37.20Bb	31.20Bb	30.80Bb	33.90Bb				
ADIP (g/kg DM)	CK	29.71Aa	23.15Ab	25.78Ab	23.58Ab	24.74Ab	0.96	0.029	<0.01	<0.01
	PEG	12.61Ba	14.68Ba	12.68Ba	12.70Ba	14.78Ba				
NPN (% TN)	CK	36.02Bb	44.13Aa	46.10Ba	40.95Ba	43.56Ba	1.718	<0.01	<0.01	<0.01
	PEG	53.70Ad	58.33Acd	62.29Ab	63.54Aab	69.44Aa				
AN (% TN)	CK	1.48Bc	2.53Bb	2.96Bb	3.63Ba	4.05Ba	0.240	<0.01	<0.01	<0.01
	PEG	3.17Ad	3.80Ad	4.63Ac	5.59Ab	7.33Aa				
AA-N (% TN)	CK	9.93B	10.67B	11.23B	12.72B	16.43Ba	1.65	<0.01	<0.01	<0.01
	PEG	26.25Ab	29.14Ab	34.98Aa	33.04Aa	35.50Aa				
Peptide-N (% TN)	CK	24.63	30.96	31.98	24.68	22.50	0.72	>0.05	>0.05	<0.01
	PEG	24.27	25.41	22.69	25.78	25.11				

^1^*n* = 3.

CK, control; PEG, polyethylene glycol; SOLP, soluble protein; NDIP, neutral detergent-insoluble protein; ADIP, acid detergent-insoluble protein; NPN, non-protein nitrogen; AN, ammonia nitrogen; AA-N, amino acid-nitrogen.

^*A,B*^Means in the same column followed by different uppercase letters differ (*p* < 0.05); ^*a–c*^means in the same row followed by different lowercase letters differ (*p* < 0.05).

### Protein fractions of sainfoin silage

**FIGURE 1:**
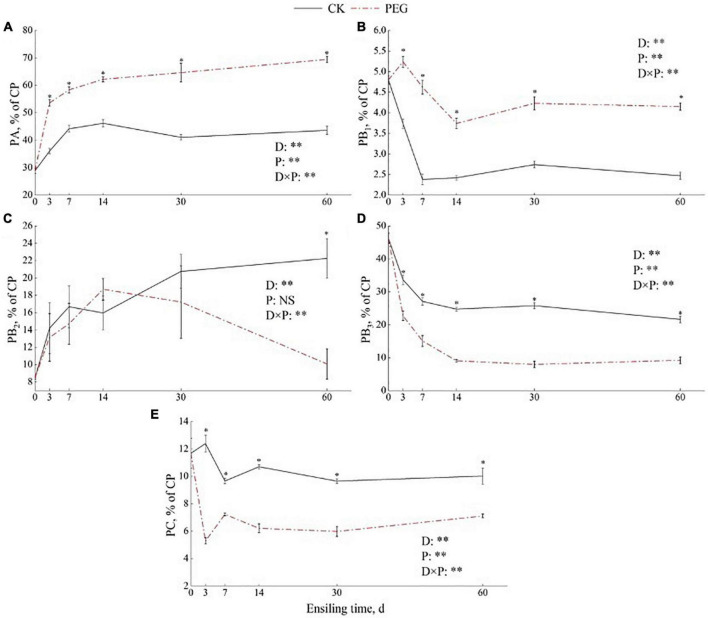
Effect of intrinsic tannins on protein fraction of **(A)** PA, **(B)** PB_1_, **(C)** PB_2_, **(D)** PB_3_, and **(E)** PC during sainfoin ensiling. PA (% CP) = NPN (% SOLP) × 0.01 × SOLP (% CP); PB_1_ = SOLP (% CP)-PA (% CP); PB_2_ = 100-PA (% CP)-PB_1_ (% CP)-PB_3_ (% CP)-PC (% CP); PB_3_ = NDIN (% CP)-ADIN (% CP); PC = ADIN (% CP). PEG, polyethylene glycol. D = effects of ensiling days; P = effects of addition with PEG. D × P = effects of interaction between ensiling days and PEG treated. Error bars mean SEM. Asterisks (*p < 0.05, ^**^p < 0.01) indicate that there was a significant difference between the control and PEG-treated groups.

The protein fraction dynamics during sainfoin ensiling determined based on the CNCPS system are shown in Figures 1A–E. PA contents increased rapidly during 7 days of ensiling in both the control and PEG-treated groups. The PEG-treated groups had the highest PA contents during the entire ensiling process (*p* < 0.05). PB_1_ content decreased to 49.61% of the initial contents after 7 days of ensiling and then stabilized over time in the control sainfoin silage. However, the PEG-treated groups still had 86.42% of the initial PB_1_ content after 60 days of ensiling. In addition, PEG-treated groups had higher PB_1_ contents than the control group during the entire ensiling process (*p* < 0.05). However, PB_2_ content was higher in the control group than in the PEG-treated group after 60 days of ensiling, which were 22.26 (% CP) and 10.08 (% CP), respectively. PB_3_ content decreased rapidly during 14 days of ensiling in both the control and PEG-treated silage. PB_3_ content was higher in the control group than in the PEG-treated group during the entire ensiling process (*p* < 0.05). After 60 days of ensiling, PB_3_ content in the control was 134% higher than that in the PEG-treated group. PC content was higher in the control group than in the PEG-treated group after 60 days of ensiling, which were 10.02 (% CP) and 7.11 (% CP), respectively.

### Protease activity in sainfoin silage

Protease activity during sainfoin ensiling is presented in Table 2. PEG addition only increased AP and ACP activities after 3 days of ensiling (*p* < 0.05), which increased by 188.99% and 194.13%, respectively, when compared with the levels in the control. The PEG-treated group exhibited the highest CP activity at 7–60 days of ensiling (*p* < 0.05), which was 38.23–69.43% of the protease activity at 0 day. AP and ACP activities both increased with an increase in the ensiling period in the control group (*p* < 0.05). In the PEG treatment, both AP and ACP activities were the highest after 14 days of ensiling; however, they decreased after 60 days of ensiling (*p* < 0.05). CP activity did not change with change in the ensiling period in the control group (*p* > 0.05); however, CP activity increased after 30 days of ensiling in the PEG-treated group (*p* < 0.05).

**TABLE 2:** Effects of intrinsic tannins on protease activity during sainfoin ensiling.

Protease	Treatment	Ensiling days						SEM	Treatment	Day	D × T
		0	3	7	14	30	60				
APs	CK	35.68	25.89Bc	32.26Abc	76.95Aa	58.15Aab	65.24Aa	4.00	*p* < 0.05	*p* < 0.05	*p* < 0.05
	PEG	–	74.82Aab	47.51Ab	94.68Aa	73.40Aab	58.51Ab				
ACP	CK	22.54	35.58Bb	88.60Aa	92.09Aa	88.14Aa	72.33Aa	4.42	*p* < 0.05	*p* < 0.05	*p* < 0.05
	PEG	–	104.65Ab	111.40Ab	118.60Aa	98.60Ab	81.16Ab				
CPs	CK	56.12	18.62Aa	16.60Ba	18.11Ba	21.64Ba	24.65Ba	3.37	*p* < 0.05	*p* < 0.05	*p* < 0.05
	PEG	–	32.20Abc	38.23Abc	45.78Ac	69.43Aa	58.36Aab				

*n* = 3.

CK, control; PEG, polyethylene glycol; Aps, aminopeptidases (protease activity proportions of day 0, %, units/h/DM); CPs, carboxypeptidases (protease activity proportions of day 0, %, units/h/DM); ACP, acid protease (protease activity proportions of day 0, %, units/h/DM).

^*A,B*^Means in the same column followed by different uppercase letters differ (*p* < 0.05); ^*a–c*^means in the same row followed by different lowercase letters differ (*p* < 0.05).

### Bacterial community structure in sainfoin silage

**FIGURE 2:**
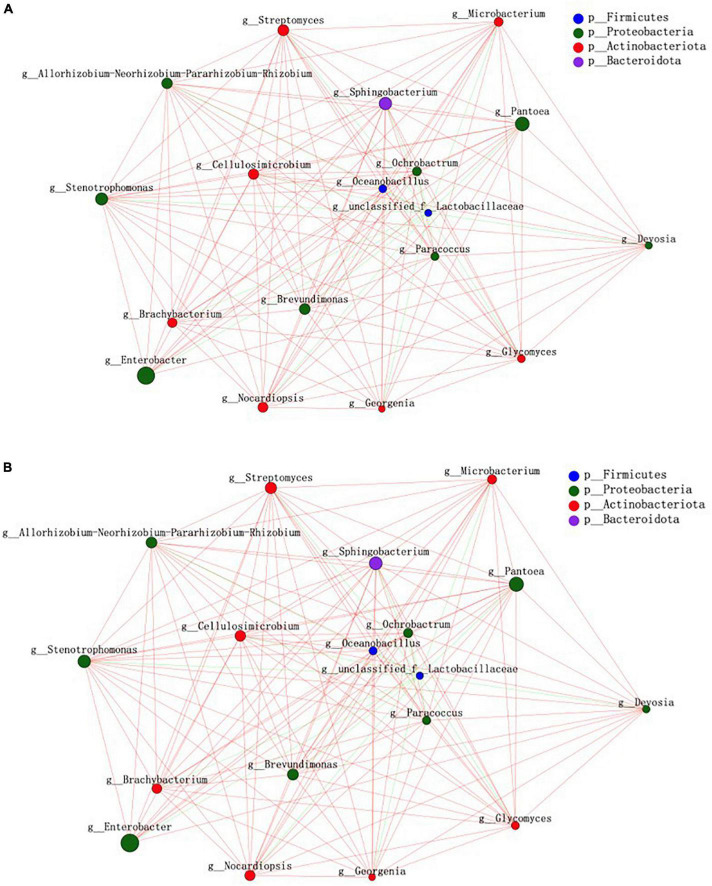
Bacterial community structure in sainfoin silage. **(A)** Bacterial network analysis of sainfoin silage in the control. **(B)** Bacterial network analysis of sainfoin silage in the PEG-treated group.

Bacterial community network analysis results are illustrated in Figures 2A,B, based on betweenness centrality, degree centrality, and closeness centrality values. In the control treatment, the keystone bacteria were *Sphingobacterium, Stenotrophomonas, Pantoea*, and *Cellulosimicrobium*. In the PEG treatment group, the keystone bacteria were *Microbacterium, Enterococcus, Sphingobacterium*, and *Brevundimonas*.

### Metabolomic profiles

**FIGURE 3:**
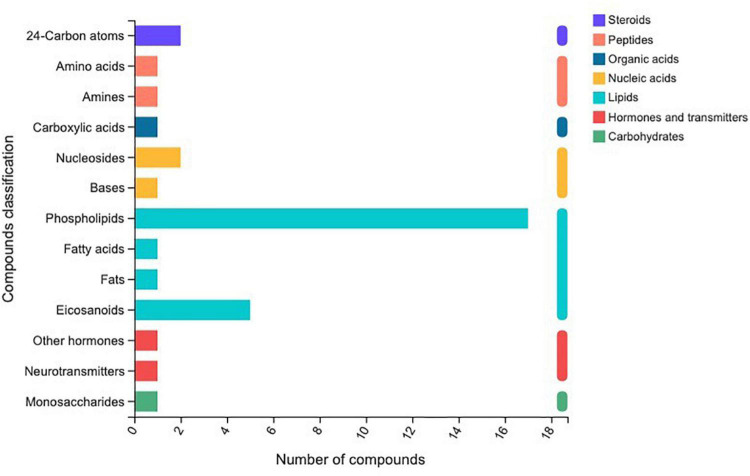
Different metabolites annotated by Kyoto Encyclopedia of Genes and Genomes in sainfoin silage.

**FIGURE 4:**
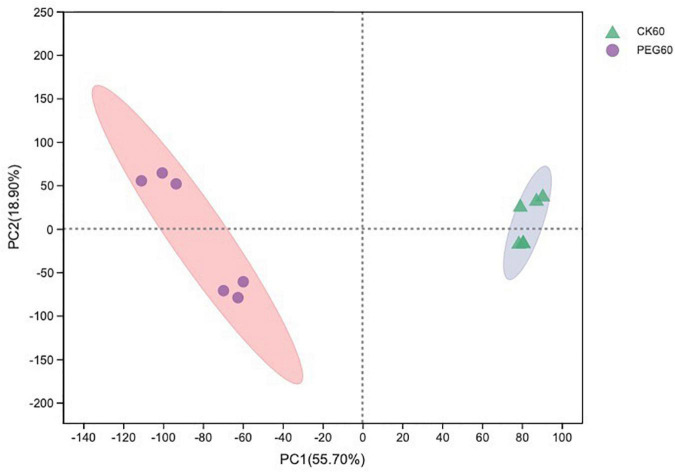
The principal component analysis (PCA) score plot of the metabolites in sainfoin silage (CK 60, control group after 60 days of ensiling; PEG 60, PEG-treated group after 60 days of ensiling).

**FIGURE 5:**
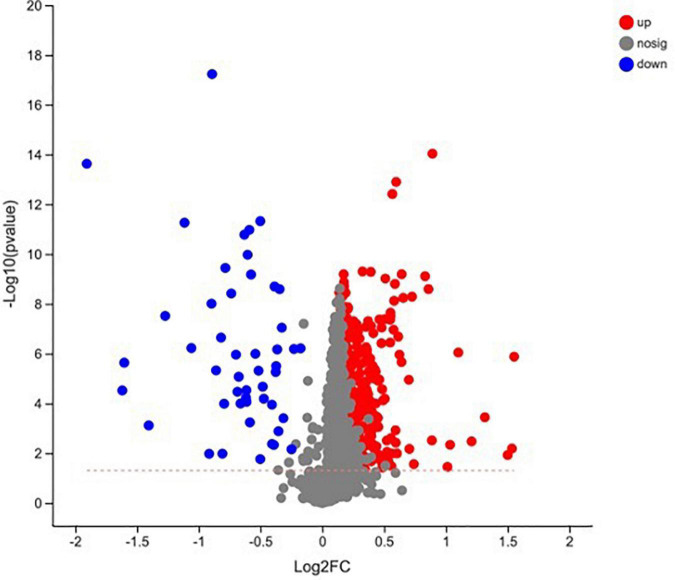
Volcano plot analysis of the metabolites in sainfoin silage (CK 60, control group after 60 days of ensiling; PEG 60, PEG treated group after 60 days of ensiling).

**FIGURE 6:**
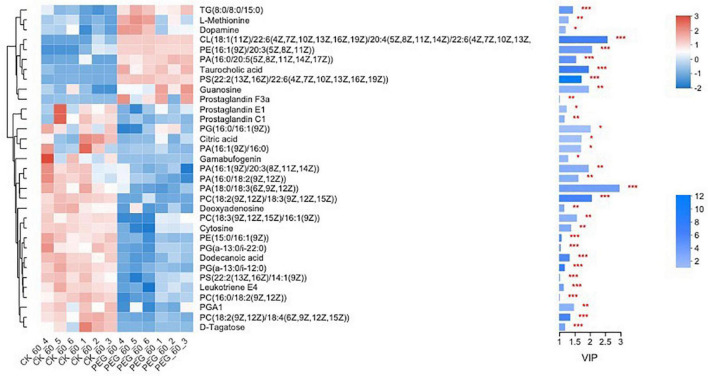
Heatmap of the differentially accumulated metabolites in sainfoin silage (CK 60, control group after 60 days of ensiling; PEG 60, PEG treated group after 60 days of ensiling). *p < 0.05, ^**^0.01 < *p* < 0.05, ^***^p < 0.01.

**FIGURE 7:**
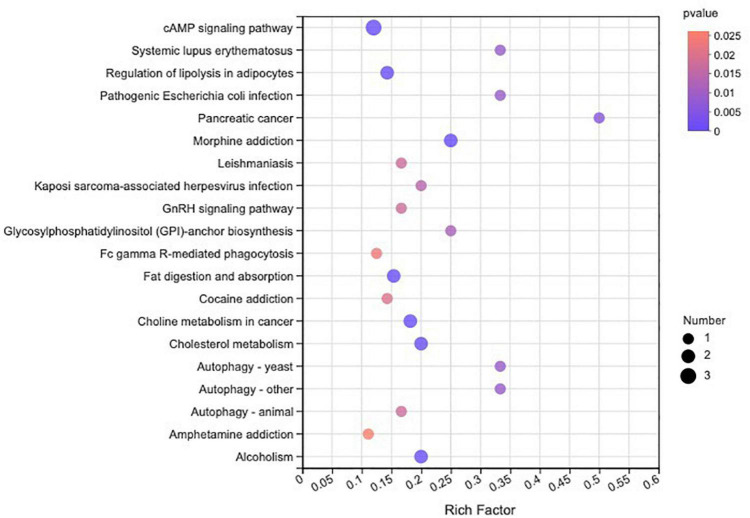
Kyoto Encyclopedia of Genes and Genomes pathway enrichment analysis for differentially accumulated metabolites in sainfoin silage.

In total, 510 metabolites (Supplementary Table 1) were identified after 60 days of sainfoin ensiling, among which 33 metabolites were annotated based on the KEGG database, as shown in Figure 3. The metabolites consisted of lipids, nucleic acids, organic acids, peptides, carbohydrates, hormones and transmitters, and steroids. Lipids were the most abundant metabolites (72%) among the 33 metabolites. There were clear distinctions in metabolites between the control and the PEG-treated group based on the PCA and volcano plot analysis results (Figures 4, 5). Based on specific screening conditions (VIP > 1, *p* < 0.05), 10 upregulated metabolites and 23 downregulated metabolites were identified in the PEG-treated group when compared with the control after 60 days of ensiling, as shown in Figure 6. Among the upregulated metabolites, phospholipids (four metabolites, including cardiolipin, phosphatidylethanolamine, phosphatidylserine, and phosphatidate) were the most abundant, followed by L-methionine, prostaglandin F3a, dopamine, guanosine, taurocholic acid, and triacylglycerol. Similar trends were observed with regard to the upregulated metabolites, with 13 metabolites belonging to phospholipids (such as phosphatidate, phosphatidylcholine, phosphatidylglycerol, and phosphatidylserine) followed by eicosanoic acids (four metabolites, namely, 15-hydroxy-9-oxoprosta, prostaglandin C1, leukotriene E4, and prostaglandin E1), two nucleic acids (cytosine and deoxyadenosine), and four other metabolites (gamabufogenin, citric acid, D-tagatose, and dodecanoic acid). The most affected pathways between the control and PEG-treated groups were “cAMP signaling” (three metabolites were associated with the “cAMP signaling” pathway), followed by “regulation of lipolysis in adipocytes,” “morphine addiction,” “fat digestion and absorption,” “choline metabolism,” “cholesterol metabolism,” and “Alcoholism” with two metabolites were associated with the “Alcoholism” pathway (Figure 7).

### Correlation between bacteria relative abundance and metabolites

**FIGURE 8:**
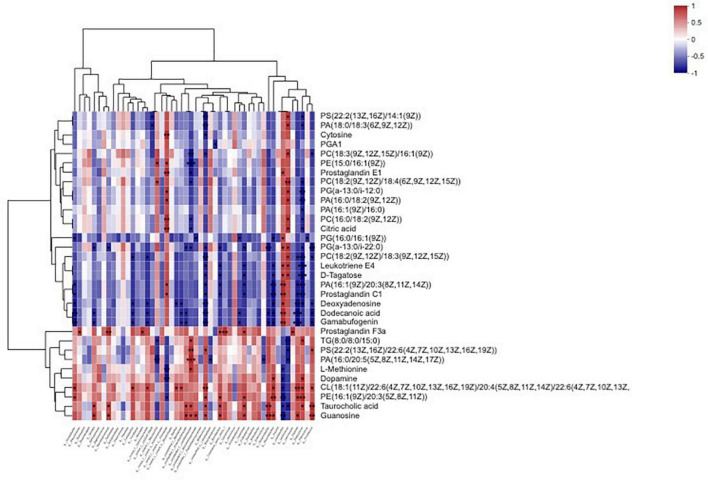
Heatmap of the correlation analysis for bacteria and metabolites in sainfoin silage. *p < 0.05, ^**^0.01 < *p* < 0.05, ^***^p < 0.01.

The results of correlation analysis between bacterial abundance and metabolites in sainfoin silage are shown in Figure 8 and Supplementary Table 3. There was no significant correlation between *Enterobacter* abundance and metabolites (*p* > 0.05). *Lactobacillus* were significantly and highly positively correlated with prostaglandin E1, phosphatidylglycerol (two metabolites), leukotriene E4, D-Tagatose, phosphatidate, prostaglandin C1, deoxyadenosine, dodecanoic acid, and gamabufogenin (*R*^2^ = 0.8286, 0.8286, 0.8286, 0.8286, 0.8286, 0.9429, 0.9429, 0.8857, 0.9429, and 0.9429, respectively, *p* < 0.05), and were significantly negatively correlated with L-methionine, dopamine, cardiolipin, phosphatidylethanolamine, taurocholic acid, and guanosine (*R*^2^ = −0.8286, −0.8857, −0.8857, −0.9429, −0.8286, and −0.9429, respectively, *p* < 0.05). *Pediococcus* were significantly negatively correlated with phosphatidylserine, phosphatidate (four metabolites), phosphatidylcholine (three metabolites), phosphatidylglycerol (two metabolites), citric acid, leukotriene E4, D-Tagatose, prostaglandin C1, and deoxyadenosine (*R*^2^ = −0.8286, −0.8286, −0.8857, −0.9429, −0.9429, −0.8286, −0.8286, −0.8286, −0.8286, −0.9429, −1, −1, −0.9429, −0.9429, and −0.8857, respectively, *p* < 0.05), and were significantly positively correlated with triacylglycerol, dopamine, cardiolipin, phosphatidylethanolamine, and taurocholic acid (*R*^2^ = 0.8857, 0.8857, 0.8857, 0.9429, and 0.8286, respectively, *p* < 0.05). *Weissella* was significantly positively correlated with prostaglandin F3a (*R*^2^ = 0.8857, *p* < 0.05).

Three keystone bacteria were correlated with metabolites, including *Enterococcus, Stenotrophomonas*, and *Pantoea*. *Enterococcus* was significantly negatively correlated with one metabolite, phosphatidate (one metabolite (*R*^2^ = −0.829, *p* < 0.05). *Stenotrophomonas* was significantly positively correlated with prostaglandin F3a, taurocholic acid, and guanosine (*R*^2^ = 0.9411, 0.8197, and 0.8197, respectively, *p* < 0.05). *Pantoea* was significantly negatively correlated with phosphatidylcholine, phosphatidate, prostaglandin C1, deoxyadenosine, dodecanoic acid, and gamabufogenin (*R*^2^ = −0.8286, −0.8857, −0.8857, −0.9249, −1, and −1, respectively, *p* < 0.05), and were significantly positively correlated with cardiolipin, phosphatidylethanolamine, and guanosine (*R*^2^ = 0.9249, 0.8857, and 0.8857, respectively, *p* < 0.05).

## Discussion

### Nitrogen distribution and protein fractions in sainfoin silage

Proteolysis in silage generally takes place *via* two steps. First, plant proteases hydrolyze proteins into peptides and free amino acids (FAA); afterward, further degradation by microbial activity occurs, usually mediated by *Clostridium* and *Enterobacter* (McDonald et al., 1991). Usually, *Clostridium* is more active in moist environments with moisture levels >75% (Muck, 2010). However, in a previous study, no *Clostridium* activity was detected during sainfoin silage with a DM content of 23% (Huang et al., 2022). As a direct indicator of protein hydrolysis during ensiling, NPN consists of peptides, FAA, and NH_4_^+^-N (Licitra et al., 1996). According to the results of the present study, NPN content in the control treatment stabilized after 7 days of ensiling; however, in the PEG-treated group, NPN increased with an increase in ensiling time. The results suggest that CT decreased protein degradation during the early silage fermentation stage. AN contents in silage, which are produced by both plant proteases and microbial activities, are generated from protein hydrolysis (Li X. et al., 2018). A previous study observed that PEG addition had no effect on *Enterobacter* relative abundance during the entire process of sainfoin ensiling (fermentation 60 days); however, AN content was the highest in PEG-treated sainfoin silage during the entire process of sainfoin ensiling (fermentation 60 days). Furthermore, AN content increased in both the control and PEG-treated group with an increase in ensiling time (Huang et al., 2022). The results suggested that *Enterobacter* relative abundance may not influence AN content in sainfoin silage. However, NH_4_^+^-N content in silage formed by deamination from FAA is mainly attributed to bacterial enzyme activity rather than plant enzyme activity (McDonald et al., 1991). Metagenomic analysis revealed that NH_4_^+^-N contents were associated with nitrite reductase, with the gene encoding nitrite reductase mainly assigned to *Enterobacter* (Li R.R. et al., 2021). Considering the present results, AA contents were lower in the control group than in the PEG-treated group during the entire process. The higher AN contents in the PEG-treated silage were likely due to higher AA contents than in the control group. PEG addition increased NH_4_^+^-N content significantly during the entire ensiling process (*p* < 0.05). Other researchers have observed that PEG causes high levels of buffer soluble N in silage, which is attributed to the strong tannin-protein binding effect of CT in sainfoin (Lorenz et al., 2010). Compared to other tannin-rich legume forage, CT from sainfoin may have the lowest NH_4_^+^-N levels in silage, which confirms the capacity of CT from sainfoin to strongly inhibit proteolysis during ensiling (Brinkhaus et al., 2017). In addition, according to Huhtanen et al. (2002), high-quality grass silage has AN contents that account for <5% of the total N. In the present study, NH_4_^+^-N contents in the control (4.0% of total N) were less than those in the PEG-treated group (7.3% of total N) after 60 d. Therefore, CT from sainfoin could facilitate the maintenance of high-quality sainfoin silage. Among the N distributed in silage, N in peptides may be more useful for rumen microbial N synthesis, as a previous *in vitro* study observed that certain bacteria from rumen utilize N from peptides more efficiently than N from FAA or from ammonia (Cruz Soto et al., 1994).

According to the results of the present study, there were no significant differences between the control and PEG-treated groups during the entire ensiling process (*p* > 0.05). However, in the case of *Megasphaera elsdenii* from the rumen, which is one of the main lactate-fermenting rumen bacterial species, AA N would be more useful than peptide N (Nsereko et al., 1998). PEG-treated silage had higher AA contents than the control silage over the entire ensiling process in the present study. Both the control and PEG-treated groups had the highest AA contents after 60 days of ensiling, which were 16.43 and 35.50%, respectively. The results suggest that PEG addition increased AA contents during sainfoin ensiling, which may promote the growth of certain bacteria in the rumen, with no effects on peptide N. The increased susceptibility of protein to degradation following PEG addition could be attributed to the high capacity of PEG to bind tannins, which could cause either an exchange of tannin-bound protein with PEG or prevent tannin-bound protein formation (Lorenz et al., 2010). In addition, microflora composition during ensiling is influenced by AAs, such as arginine, particularly when pH is not decreased considerably (Winters et al., 2000). In a previous study, PEG addition increased lactic acid bacteria (LAB) activity (*Pediococcus* relative abundance) during ensiling (Huang et al., 2022). Hence, the inhibition of LAB activity by CT from sainfoin is partly attributable to decreased AA contents.

Remarkably, PEG addition increased the SOLP content, while decreasing NDIP and ADIP contents during the entire ensiling process. Similar results were observed by Peng et al. (2018), although NDIP content remained unchanged in their study. In addition, in the present study, the PB_3_ fraction decreased with PEG addition, and similar results were observed by Brinkhaus et al. (2017), where the protein from the PB_1_ and PB_2_ fraction shifted to the PB_3_ and PC (insoluble in acid detergent protein) fractions during sainfoin ensiling, suggesting that CT can decrease protein degradation during ensiling.

### Protease activity in sainfoin silage

During ensiling, proteolysis can occur in the absence of microbial growth through sterilized herbage, which suggests that plant enzymes are the main agents driving degradation during proteolysis (McKersie and Buchanan-Smith, 1982). ACP, CPs, and APs are three major proteases formed during ensiling; CP and ACP exhibit activities at pH 5.2, ACP exhibits optimal activity under a silage pH of 4.5, whereas APs lose their function (McKersie, 1981). In the present study, sainfoin silage pH was 4.5–4.69 at 3–30 days (pH results can find at Huang et al., 2022); however, the APs still exhibited 25.89 to 94.68% activity when compared to 0 day protease activity. A previous study reported that CP and AP activities stabilized at 14 days but intensified after 30 days of ensiling; however, the pH decreased from 4.1 to 3.9 over a 30-day ensiling period (He et al., 2020). Several studies have suggested that proteolysis still takes place at pH <4.0 (Carpintero et al., 1979; Henderson, 1993). Hence, the silage environment, in which APs maintain activity at pH <4.1 during ensiling, could be much more complex than the herbage incubation environment. Furthermore, PEG had no effect on pH when compared with the control (pH results can find at Huang et al., 2022); however, the PEG group exhibited the highest AP and ACP activity after 3 days of ensiling. Considering there was no difference in pH between the PEG-treated and control groups, the difference in protease activity was mainly attributed to inhibition by CT. The results suggest that CT inhibit AP and ACP activity only in the early fermentation stages. Additionally, CT inhibited CP activity at 7–60 days of ensiling. However, He et al. (2019) observed that CT from *Neolamarckia cadamba* leaf (NCL) had no effect on CP activity during 30 days of ensiling. The mechanisms *via* which CT inhibit protease activity, such as tyrosinase, including competition with substrates for active enzyme sites and binding to the enzyme substrate complex, which occur through hydrogen bonding and hydrophobic interactions (Song et al., 2020). Several factors, such as molecular size, mean degree of polymerization (mDP), presence of galloyl groups, and interflavan-3-ol linkages, influence CT affinity for proteins (Zeller, 2019). Previous studies have reported that CT from different materials, such as *Rhododendron pulchrum* leaves (Chai et al., 2015), sour jujube (*Ziziphus jujuba* Mill. var. *spinosa* [Bunge] Hu ex. H. F. Chow) (Song et al., 2020), and *Pinus thunbergii* needles (Yang et al., 2021) show varied ability to inhibit tyrosinase activity, with IC_50_ values ranging from 43.7 to 145.35 μg/ml. Therefore, the ability of CT from sainfoin to inhibit protease activity during ensiling might vary from that of NCL.

*Cellulosimicrobium* is a Gram-positive anaerobic bacteria that secretes several enzymes, such as glycosidases, galactosidases, glucosidases, and esterases; it exploits different types of carbon sources (arabinose, xylose, glucose, etc.) to produce organic acids, in addition to being able to secrete nitrate reductase, pyrazinamidases, and pyrrolidine acyl aromatic amide enzymes (Hamada et al., 2016). *Cellulosimicrobium* became keystone bacteria in silage result as all of these enzymes could be involved in different metabolic pathways. In the present study, *Cellulosimicrobium* did not represent keystone bacteria in the PEG-treated silage. The difference could be partly attributed to CT combining with protein and inhibiting enzyme activity.

### Bacteria community in sainfoin silage

Keystone bacteria in microecological environments are increasingly attracting the attention of researchers, supported by the proliferation of high-throughput sequencing technologies. Microbial community structure and function regulation by keystone bacteria is not associated with their relative abundance. On the contrary, bacteria with low relative abundance can often be keystone bacteria, and keystone bacteria can selectively regulate microbial community structures (Banerjee et al., 2018). In the present study, PEG addition altered the distribution of keystone bacterial communities in silage significantly. The results suggested that CT had a significant effect on keystone bacterial community composition during sainfoin ensiling. In the present study, *Sphingobacterium* were the keystone bacteria in both the control and PEG-treated groups. *Sphingobacterium* were also abundant in fresh corn silage and barley silage, especially in corn silage (Xu et al., 2019; Liu et al., 2020); however, the relative abundance of *Sphingobacterium* decreased rapidly to below 1% and may participate in ethanol production during ensiling (Ni et al., 2017). Additionally, *Sphingobacterium* is involved in biotic carbon dioxide (CO_2_) sequestration as it was negatively correlated with CO_2_ production during ensiling (Chen et al., 2021). Similar to *Sphingobacterium*, during anaerobic autotrophic growth, *Stenotrophomonas* can convert CO_2_ into organic acids through microbial electrosynthesis (Bian et al., 2020). Both the bacteria could use different WSCs to produce organic acid (Palleroni and Bradbury, 1993). Therefore, the two bacteria could compete with LAB during ensiling. However, a previous study observed that the presence of *Sphingobacterium* and *Stenotrophomonas* in silage was associated with increased richness and relative abundance of LAB in a bacterial community (Li M. et al., 2021). The difference may be attributed to the relative abundance of keystone bacteria being the lowest when compared with LAB results as competition for WSC is weakened; however, keystone bacteria regulate microbial activity *via* different metabolic pathways. Ogunade et al. (2018) first observed that *Pantoea* was significantly negatively corrected with AN in alfalfa silage. Several studies have reported similar results in silage (Dong et al., 2020; Yang et al., 2020). High *Pantoea* relative abundance was observed in silage but it decreased by a treated group company, with butyrate acid contents reduced. A study on corn silage indicated that *Pantoea* was more active under high moisture contents, with a positive correlation with butyrate acid contents (Guan et al., 2018). Such studies indicate that the metabolic pathway of *Pantoea* is more complex, which was attributed to the capacity of *Pantoea* not only to convert WSC to organic acid but also to decrease AN content in silage. Therefore, considering the low *Pantoea* relative abundance when compared with LAB, there could be different mechanisms of reducing pH during ensiling between *Pantoea* and LAB. A study on sainfoin and alfalfa silage mixture indicated that *Pantoea* relative abundance increased with an increase in the proportion of sainfoin in the mixture (Wang et al., 2021), which could be related to CT. In the present study, *Pantoea* was not among the keystone bacteria in the PEG-treated silage. Hence, CT might affect keystone bacteria community structure through its effect on *Pantoea* during sainfoin ensiling. *Microbacterium* exists in fresh corn with a relative abundance of 3.5–6.35%, which decreases considerably with a prolongation of ensiling time (Xu et al., 2020b). *Microbacterium foliorum* could use fructose during ensiling (Wang et al., 2020). To the best of our knowledge, there are few studies on *Microbacterium* activity during ensiling. Therefore, further studies on the mechanism *via* which *Microbacterium* become keystone bacteria during sainfoin ensiling are required.

*Enterococcus* exhibit greater activity during the early fermentation stages of silage, with *Leuconostocs, Pediococci*, and *Lactococci* involved in the initiation of silage fermentation through lactic acid production; however, all the bacteria above are replaced gradually by *Lactobacillus* with the prolongation of ensiling time (Cai and Benno, 1998). Additionally, *Enterococcus* could transform FAA to produce AN and bioamines through different metabolic pathways through corresponding deaminases and decarboxylases (Oladosu et al., 2016); for example, lysine decarboxylase forms cadaverine, tyrosine decarboxylase forms tyramine, and arginine decarboxylase forms arginine (Li R.R. et al., 2021). Furthermore, AN synthesis involves different metabolic pathways and enzymes, such as nitrite reductase, nitrogenase, and hydroxylamine reductase (Huang et al., 2020). In the present study, *Enterococcus* was not among the keystone bacteria in the control attributed to the deactivation of enzymes through that action of CT in combination with protein.

## Metabolome profile of sainfoin silage

A total of 510 metabolites (Supplementary Table 1) were identified after 60 days of sainfoin ensiling, with 300 metabolites distinct between the CK and PEG-treated groups. The most distinct metabolites were phospholipids (17 metabolites), followed by eicosanoids (5 metabolites), nucleosides and 24-carbon atoms (5 metabolites), and others (1 metabolite). Several studies have observed that catechins inhibit bacteria by damaging the lipid bilayer membrane (Tsuchiya, 2001; Nakayama et al., 2002; Caturla et al., 2003), *via* hydrogen bonding between their polyhydroxyl groups and phosphocholine moiety, and interaction with phospholipids (Li Z. et al., 2018). Considering CT contains four types of catechins (Zeller, 2019), the results suggest that CT from sainfoin has strong effects on bacterial phospholipid synthesis. The PCA analysis results revealed distinct metabolites between the control and PEG-treated groups. However, a previous study showed that control and PEG-treated groups had similar microbial community structures at the genus level (Huang et al., 2022). Therefore, the main reason for the variation could be attributed to CT, which had a strong effect on microbial metabolism during ensiling.

One amino acid (L-methionine) was upregulated following PEG addition, whereas citric acid was downregulated following PEG. Furthermore, two nucleosides, including cytosine and deoxyadenosine, were downregulated and one nucleoside, guanosine, was upregulated following PEG addition. Catechin also inhibits bacteria *via* DNA degradation, and it is more toxic to Gram-positive bacteria than Gram-negative bacteria (Fathima and Rao, 2016). Enterobacter, a Gram-negative bacteria, was not affected by CT during sainfoin ensiling, but *Pediococcus*, a Gram-positive bacteria, was highly inhibited by CT during sainfoin ensiling (Huang et al., 2022). The results suggest that CT affected Gram-positive bacteria through inhibition of phospholipid activity and gene synthesis during ensiling.

During ensiling, various metabolites are produced through complex metabolic reactions, which are regulated by different metabolic pathways (Xu et al., 2020a). The most affected metabolic pathways were annotated to the “cAMP signaling pathway,” with five phospholipids, deoxyadenosine, and dopamine associated with the pathway, after 60 days of sainfoin ensiling. Furthermore, sainfoin ensiling could alter the metabolome, including altering the dynamics of isoflavone, proanthocyanidins, polyphenol, and so on, through the flavonoid and isoflavonoid biosynthesis pathways. However, inoculation of sainfoin with LAB eliminated the effects, suggesting that LAB inoculation could mainly affect the flavonoid and isoflavonoid biosynthesis pathways (Xu et al., 2020a). In the present study, PEG addition had no effect on the flavonoid biosynthesis pathway (Supplementary Table 2), which indicates that CT biosynthesis is mainly affected by the ensiling process, as CT consists of flavan-3-ol subunits (Zeller, 2019).

In the present study, PEG addition led to the upregulation of 10 metabolites and downregulation of 23 metabolites. *Lactobacillus* were significantly negatively correlated with six metabolites among the upregulated metabolites and were significantly positively correlated with 10 metabolites among the downregulated metabolites. On the contrary, *Pediococcus* was significantly positively correlated with five metabolites among the upregulated metabolites, and significantly negatively correlated with 15 metabolites among the downregulated metabolites. Additionally, phospholipids were the most affected metabolites and were correlated with both *Lactobacillus* and *Pediococcus*. The results suggested the antagonistic effects between *Lactobacillus* and *Pediococcus* during ensiling. Among the metabolites upregulated following PEG addition, dopamine is converted by tyrosine. *Pediococcus* was significantly positively correlated with dopamine, but *Lactobacillus* was significantly negatively correlated with dopamine. The cell-free supernatants of some *Pediococcus* (*Pediococcus acidophilus*) have stronger stimulation effects on dopamine production by *Enterobacter* (*Escherichia coli*) than *Lactobacillus* (*Lactococcus lactis* subsp. *lactis*) (Ozogul et al., 2017). According to the results, PEG upregulated dopamine after 60 days of sainfoin ensiling, which was correlated with CT strongly inhibiting *Pediococcus* activity during sainfoin ensiling.

## Conclusion

Polyethylene glycol addition decreased NDIP and ADIP contents, with NPN contents increased, during sainfoin ensiling. CT from sainfoin decreased proteolysis mainly *via* the inhibition of protease activity, particularly CP activity. In addition, the decrease in proteolysis was partly attributed to CT inhibiting *Pediococcus* activity, based on the highly positive correlation between *Pediococcus* and dopamine. CT also affected bacteria community structure, potentially through shifts in keystone bacteria. The most distinct metabolites between the control and PEG-treated groups were phospholipids, which indicated that CT inhibited bacterial activity through decreased phospholipid synthesis during ensiling.

## Data availability statement

The original contributions presented in this study are included in the article/Supplementary material, further inquiries can be directed to the corresponding authors.

## Author contributions

RH: conceptualization, methodology, and writing – review and editing. FZ: conceptualization, resources, validation, and supervision. XW: resources and investigation. CM: conceptualization, methodology, and visualization. All authors have read and agreed to the published version of the manuscript.

## Footnotes

1
http://bioconductor.org/packages/release/bioc/html/ropls.html

2
https://cloud.majorbio.com

3
https://www.majorbio.com
