# Supplementary material for: Effects of intrinsic tannins on proteolysis dynamics, protease activity, and metabolome during sainfoin ensiling
Source: Front Microbiol. 2022 Aug 18;13:976118. doi: 10.3389/fmicb.2022.976118 (PMC9433569; doi:10.3389/fmicb.2022.976118)
Supplement: Supplementary file 1 [file Data_Sheet_1.docx]

1.Characteristic of sainfoin silage

The fermentation characteristic of sainfoin silage is show in Figure S1.


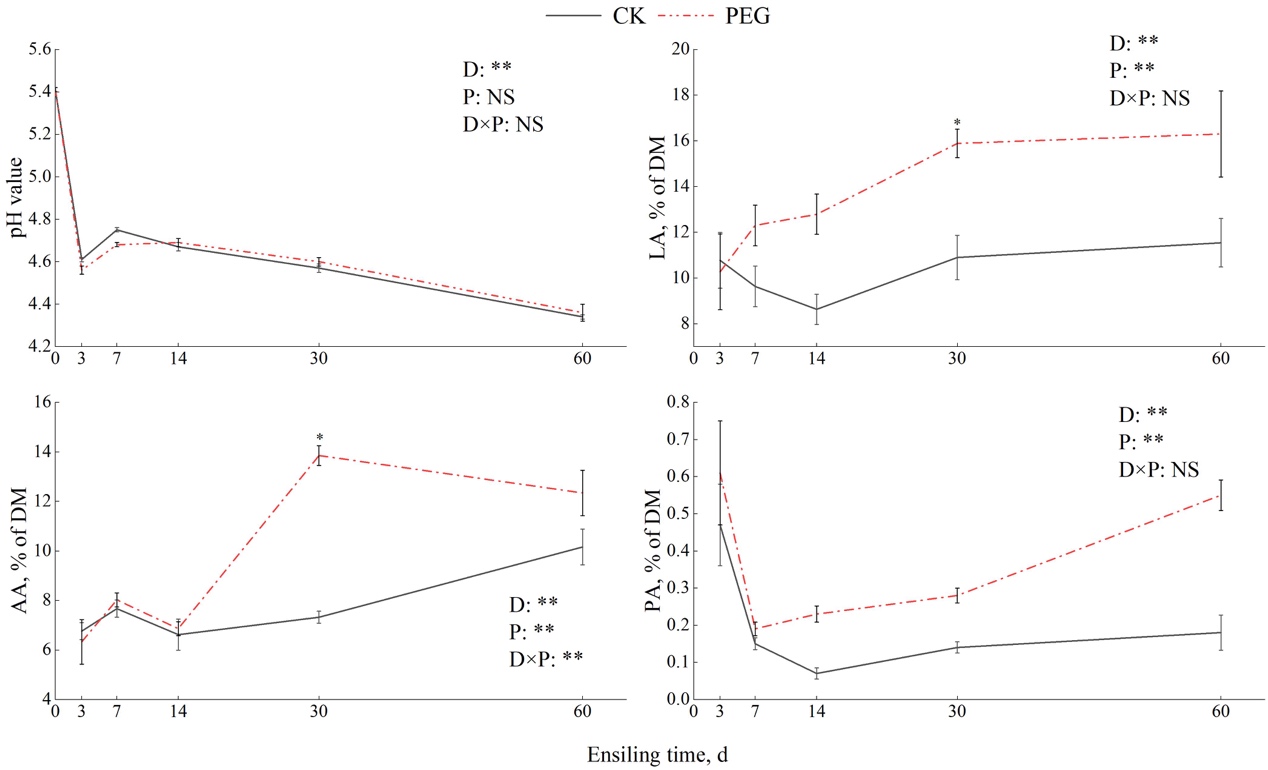


Figure S1. Effects of intrinsic tannins on fermentation characteristics of sainfoin silage. (a) PH, (b)LA, (c) AA, (d) PA. LA: lactic acid; AA: acetic acid; PA: propionic acid. PEG: polyethylene glycol. D= effects of ensiling days; P= effects of addition with PEG. D×P= effects of interaction between ensiling days and PEG treated. Error bars mean SEM. Asterisks (**P*< 0.05, ***P*< 0.01) indicate that there was significant difference between control and PEG treated groups.

Reference

Huang, R. Z., Zhang, F. F., Wang, T., Zhang, Y. L., Li, X., Chen, Y. C., et al. (2022). Effect of Intrinsic Tannins on the Fermentation Quality and Associated with the Bacterial and Fungal Community of Sainfoin Silage. *Microorganisms* 10, 844. Doi: 10.3390/ microorganisms10050844
